# A novel *TCN2* mutation with unusual clinical manifestations of hemolytic crisis and unexplained metabolic acidosis: expanding the genotype and phenotype of transcobalamin II deficiency

**DOI:** 10.1186/s12887-022-03291-5

**Published:** 2022-04-29

**Authors:** Pongpak Pongphitcha, Nongnuch Sirachainan, Arthaporn Khongkraparn, Thipwimol Tim-Aroon, Duantida Songdej, Duangrurdee Wattanasirichaigoon

**Affiliations:** Department of Pediatrics, Faculty of Medicine Ramathibodi Hospital, Mahidol University, 270 Rama 6 Rd., Rajthewi, Bangkok, 10400 Thailand

**Keywords:** Transcobalamin II deficiency, *TCN2*, Megaloblastic anemia, Bone marrow failure, Pneumocystis pneumonia

## Abstract

**Background:**

Transcobalamin deficiency is a rare inborn metabolic disorder, characterized by pancytopenia, megaloblastic anemia, failure to thrive, diarrhea, and psychomotor retardation.

**Case presentation:**

We describe a patient who first presented at 3 months of age, with pancytopenia, hepatosplenomegaly, recurrent infection, metabolic acidosis, and acute hemolytic crisis. Extensive hematologic and immunologic investigations did not identify inherited bone marrow failure syndrome, acute leukemia or its related disorders. Whole exome sequencing identified a novel homozygous *TCN2* mutation, c.428-2A > G and mRNA study confirmed an aberrant transcription of exon 4 skipping. The mutant protein is predicted to have an in-fame 51 amino acids deletion (NP_000346:p.Gly143_Val193del). The patient exhibited marked clinical improvement following hydroxocobalamin treatment.

**Conclusions:**

Transcobalamin deficiency should be investigated in infants with unexplained pancytopenia and acute hemolytic crisis with or without typical evidence of vitamin B12 deficiency.

## Background

Transcobalamin II (TC), a cobalamin binding plasma protein, facilitates cellular uptake via receptor-mediated endocytosis [1–3]. Deficiency or defect of TC, caused by *TCN2* mutations, results in intracellular cobalamin depletion that leads to a rare multisystemic disorder with autosomal recessive inheritance. Common clinical features include pancytopenia, megaloblastic anemia, failure to thrive, diarrhea, psychomotor retardation, and less frequently, immune deficiency [1]. The onset of symptoms is usually during infantile period, with the median age of 2 to 4 months [1].

Here, we reported an infant with overlapping clinical features of hematologic malignancy, inherited bone marrow failure syndrome (IBMFS), hemolytic crisis, primary immune deficiency (PID), and metabolic acidosis. The advance in next-generation sequencing had enabled the genetic diagnosis of the homozygous novel *TCN2* mutation.

## Case presentation

A 3-month-old male infant, a single child of a consanguineous Thai couple, was first presented to a local medical center with upper respiratory tract infection, anemia, petechiae, mild hepatosplenomegaly, and pancytopenia (Table 1). Acute leukemia and inherited bone marrow failure syndrome were suspected but not supported by the findings of bone marrow (BM) aspiration and biopsy, flow cytometry, and BM karyotype. At 4 to 5 months of age, the patient had two episodes of diarrhea. There was a 10% increase of circulating immature myeloid series and marked elevation of serum lactate dehydrogenase (LDH) levels, necessitating a re-evaluation for myeloid leukemia, but findings were negative.

**Table 1:** Clinical course and laboratory data including treatments and outcomes

Age (m)/ time after treatment (m)	3	4	5–7	8	9	12	14	15	15/+ 0.25	16/+ 1	17/+ 2	19/+ 4	23/+ 8
Clinical	URI	diarrhea	PJP, PEM	–	Wide gap metabolic acidosis	Anemia	Hemolytic anemia, *B. cereus* sepsis	Anemia, fatigue	More active	–	–	–	Suspected ASD
Liver/Spleen (cm)^a^	2/1	3/1	5/2	0/0	0/0	0/0	2/0	7/0	5/0	JP/0	JP/0	0/0	0/0
Red cell transfusion	✓	✓	✓	X	X	✓	✓	✓	X	X	X	X	X
Hydroxocobalamin (IM)	X	X	EN + PN^b^	X	X	X	X	1 mg daily for a week then 1 mg twice a week					
CBC													
Hb (g/dL)	3.7	5.7	8.2	10.3	14.4	8.7	3.5	9.4	9.9	10.5	12	12.3	13.7
MCV (fL)	94	77	86	98.7	99.9	102	98.8	98.4	93	90.3	82.4	75.1	82.2
RDW (%)	ND	16.3	17.4	28	15.2	16.2	26.6	31.3	22.2	19.3	15.7	13.8	13.6
WBC (10^9^/L)	4.67	2.3	20.6	7.5	14.0	6.8	14.6	7.2	8.67	9.94	9.01	12.32	9.82
N(%)	14	65	35	14	60	12	29	27	30	20	13	33	16
Platelet (10^9^/L)	25	36	29	250	338	186	164	62	331	267	261	297	285
LDH (125–220 U/L)	805	1606	610	ND	ND	1314	1786	673	365	362	275	297	288
Plasma homocysteine (5-15 μmol/L)	ND	ND	ND	ND	ND	ND	ND	53.7	1.6	2.0	2.8	2.8	2.1
Development	–	–	Roll over, Chest up, Sit with support, no babbling	–	Sit without support	Feed NG, no sucking, no babbling, stand with aid	–	Stand with aided, no babbling, no meaning word	Feed orally, babbling, increased variety of sounds, stand unaided	Walk with aided	–	Walk unaided, no meaning word	Jump, scribble, few words, no imitate, one step command with gesture

*Abbreviations*: *ASD* Autistic spectrum disorder, *CBC* Complete blood count, *EN + PN* Enteral and parenteral nutrition, *IM* Intramuscular, *JP* Just palpable, *m.* Month, *ND* No data, *NG* Nasogastric tube, *PEM* Protein energy malnutrition, *PJP* Pneumocystis jirovecii pneumonia, *URI* Upper respiratory tract infection

^a^Liver and spleen were measured expansion below the costal margin

^b^Enteral nutrition with 1 μg/day of fortified cobalamin for 2 months, parenteral nutrition with 5 μg/day of intravenous cyanocobalamin for 1 week

**Fig. 1:**
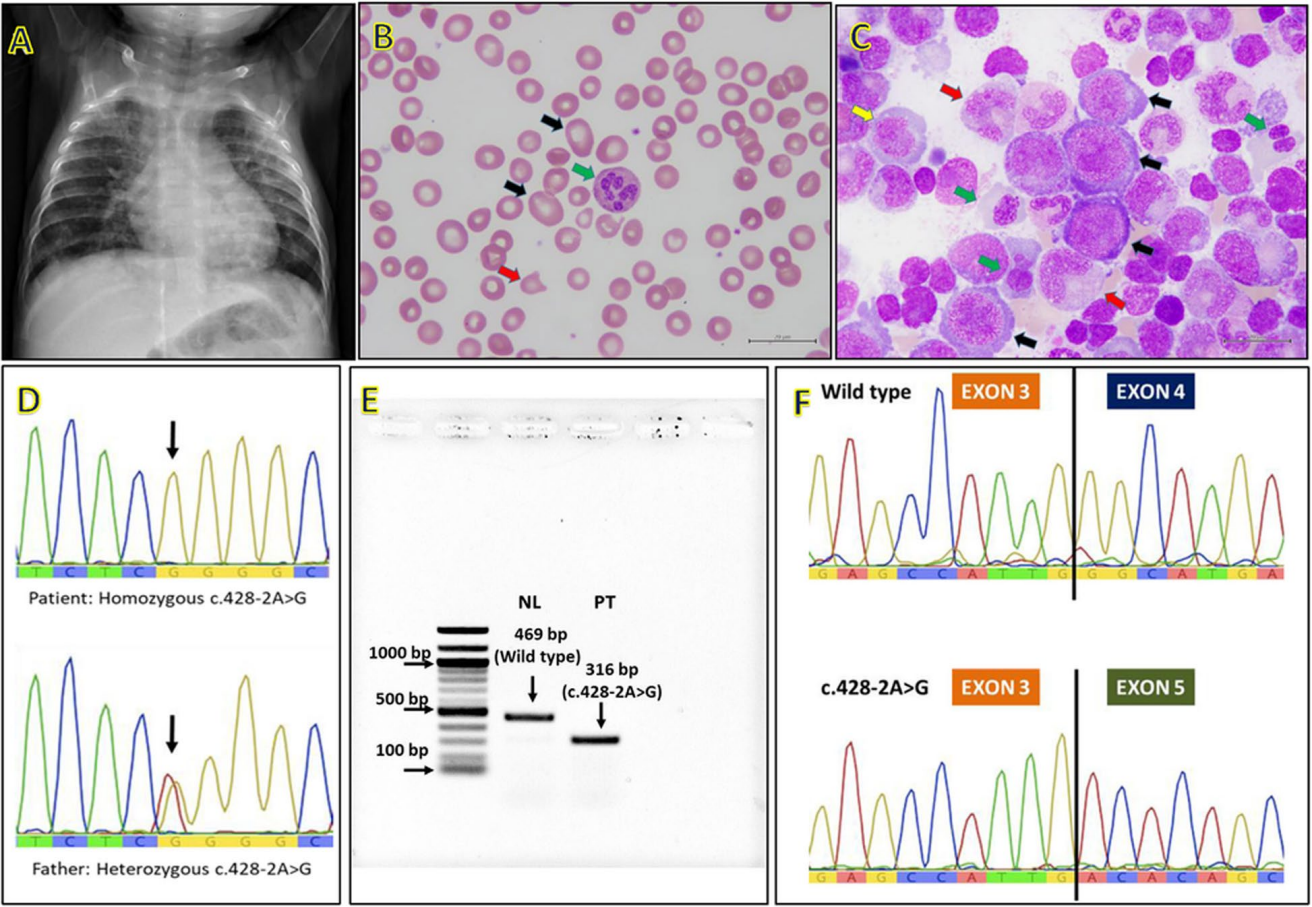
**A** Chest x-ray showing bilateral reticulonodular infiltration and hepatomegaly, **B** Peripheral blood smear (1000x) showing macro-ovalocytes (black arrow), variable-sized red blood cells as well as fragmented cell (red arrow), and hypersegmented neutrophils (green arrow). These findings give credence to megaloblastic anemia. **C** Bone marrow smear with Wright-Giemsa stain (1000x) showing megaloblastic change, large erythroblast (black arrow), nuclear and cytoplasmic maturational asynchrony of erythroid precursors (yellow arrow), dysplastic nuclei of erythroid precursors (green arrow), and giant band (red arrow). **D** Sanger sequencing, noted homozygous change from A to G at nucleotide c.428–2, and heterozygous A > G in the father (and mother: not shown). **E** mRNA (cDNA) bands showing only aberrant mRNA in the patient’s specimen (PT) and only wild type band in the normal control (NL). **F** mRNA sequencing demonstrating exon 4 skipping: coding sequence of exon 3 followed by the coding sequence of exon 5

At 5 months after arrival at our center, the patient appeared to have failure to thrive, hepatosplenomegaly, respiratory distress; and his chest X-ray indicated bilateral reticulonodular infiltration suggesting pneumonia (Fig. 1A). Bronchoalveolar lavage was performed and *Pneumocystis jirovecii* was confirmed (via PCR) as the causative pathogen. Persistent pancytopenia with monocytosis > 1000/mm^3^ was noted; thus, PID, IBMFS, myelodysplastic syndrome (MDS), juvenile myelomocytic leukemia (JMML), and autoimmune lymphoproliferative syndrome (ALPS) were investigated. The results did not support PID, including normal or slightly elevated number of absolute lymphocyte counts [CD3+, 7.3 K/μL (reference 2.2–6.4); CD4+, 4.2 K/μL (reference 1.6–4.6); CD8+, 3.1 K/μL (reference 0.7–2.4), CD19+, 0.6 K/μL (reference 0.5–1.5)] and normal or slightly increased immunoglobulin levels [IgA 1.19 mg/ml (reference 0.1–0.24), IgG 5.22 mg/ml (reference 4.57–8.83), and IgM 4.84 mg/ml (reference 0.27–0.93)]. Additional BM studies revealed normal karyotype, no canonical RAS pathway gene mutations, and normal double negative T-cell, excluding JMML and ALPS. The patient received aggressive supportive treatments including blood transfusion, parenteral nutrition and enteral tube feeding for 4 weeks plus antimicrobial agent for the pneumonia. This led to temporary improvement of hematologic profiles (Table 1). At 9 months, the patient developed mild diarrhea with inappropriate wide anion gap metabolic acidosis (serum TCO_2_ 5; gap 20), prompting investigation for inborn metabolic disorders. Acylcarnitine profile, plasma amino acids, and urinary organic acids were within normal ranges. Whole exome sequencing was performed on Illumina HiSeq2500 (Macrogen, South Korea) and analyzed following previously published protocols [4], using clinical terms of ketoacidosis (HP:0001993) and pancytopenia (HP:0001876).

At 12 months, macrocytic anemia with neutropenia was noted (Table 1 and Fig. 1B) leading to additional investigations. That revealed normal levels of serum folate (> 20; reference range 4.2–19.9 ng/ml), red-blood-cell folate (383; reference range 141–1038 ng/ml), and B12 (498; reference range 197–771 pg/ml). Serum holotranscobalamin which is an active form of vitamin B12 and an optimal marker for vitamin B12 deficiency was not measured in the present patient, owing to unavailability in Thailand. Another BM study was performed that revealed hypercellularity, megaloblastic and dysplastic changes (Fig. 1C). Targeted exome analysis of 25 genes associated with myeloid malignancy and MDS was carried out using QIAact Myeloid DNA UMI Panel (Qiagen, Hilden, Germany). Results were negative. At 14 months, the patient developed *Bacillus cereus* septicemia accompanying acute hemolytic crisis as evidenced by reticulocytosis at 22.7% (absolute count 679.6 × 10^3^/mm^3^), reduction of serum haptoglobin (< 0.025; normal range 0.32–1.97 mg/ml) and marked elevation of LDH level whilst having normal G6PD enzyme activity, hemoglobin typing, and alpha-globin gene analysis.

At 15 months of age, the results of WES became available that revealed a homozygous mutation at the acceptor site of intron 3 of *TCN2* gene, c.428-2A > G (IVS3-2A > G; NM_000355.3). Sanger sequencing confirmed the homozygous mutation in the patient and heterozygous alleles in both parents (Fig. 1D). mRNA study using previously published methods [5] demonstrated an aberrant transcript lacking exon 4 (Fig. 1E-F). Daily treatment with 1 mg of intramuscular hydroxocobalamin was initiated for 1 week, then twice a week. Serum LDH and plasma homocysteine levels were used to monitor the clinical and biochemical response, besides CBC (Table 1). The patient exhibited dramatic response to the cobalamin therapy by becoming energetic, exhibiting mouth feeding, achieving motor milestones, and requiring no further transfusion. Total plasma homocysteine rapidly declined while CBC revealed subsequent improvements in Hb, MCV, RDW and platelets. At the time of this report, the patient is 30 months-old and exhibits a mild autistic-spectrum disorder (repetitive behavior, delayed speech and poor social interaction) despite active motor development (Table 1).

## Discussion and conclusion

We describe an unusual case with complex manifestations of TC deficiency, including hepatosplenomegaly, metabolic acidosis without methylmalonic aciduria, episodic hemolytic crisis, *P.jirovecii* infection, and high LDH levels. The hepatosplenomegaly, persistent elevation of LDH levels, hypercellularity and dysplastic changes of BM led to differential diagnosis of hematologic malignancies [6–8]. These complex features complicated and delayed the diagnosis which was eventually resolved by WES. Plausible mechanisms underlying the acute hemolytic episode in this patient are intramedullary hemolysis and premature lysis of erythroid precursor cells due to ineffective hematopoiesis [8]. Hemolytic crisis is one of the characteristics described in vitamin B12 deficiency [8]. The low-to-normal MCV in the present case could be explained by frequent blood transfusion and possible low MCV of the transfused blood due to high prevalence (40%) of thalassemia carrier among Thai people including blood donors [1, 9]. By retrospective examination of the peripheral blood and BM specimens, macro-ovalocytes and hypersegmented neutrophils were seen, and giant erythroblast with nuclear cytoplasmic asynchrony and giant band were noted (Fig. 1C). In fact, these findings could be diagnostic clues for TC deficiency. We experienced that serum homocysteine and LDH levels were good alternative biomarkers in monitoring the biochemical response to cobalamin treatment in the present patient (Table 1). Normal serum cobalamin level does not exclude cobalamin deficiency and other cobalamin-related disorders, since 80% of cobalamin binds to haptocorrin, which does not represent its intracellular utilization [2, 8, 10]. It should be mentioned that homocysteine is generally undetectable or barely detectable using quantitative plasma amino acid analysis; therefore, elevated homocysteine concentration was missed. Later, when transcobalamin defect and its associated increased homocysteine level was suspected, plasma homocysteine level was measured using a separate specific test.

Less than 60 patients and 50 *TCN2* variants have been reported worldwide in which many cases were reported from regions with a high rate of consanguinity, including Turkey [1, 2, 11–14]. To our knowledge, the present patient is the first case report from a Southeast Asian country with a novel *TCN2* variant, c.428-2A > G. The variant was subsequently submitted to ClinVar database and now is accessible using a number SCV001981507 (https://www.ncbi.nlm.nih.gov/clinvar/). This mutation leads to a mutant *TCN2* transcript with deletion of 153 nucleotides (NM_000355.3:r.428_580del) and results in an in-frame deletion of 51 amino acids (NP_000346:p.Gly143_Val193del). The mutant protein is predicted to damage cobalamin transport due to loss of two of the three cobalamin binding sites, codon 152–156, 190–194, and 395–397 (www://uniprot.org) [15]. Moreover, the loss of cysteine at codon 165 could disrupt one (Cys165/Cys205) of the four disulfide bonds which are essential for maintaining the secondary structure of the TC protein [15, 16].

Cobalamin plays an important role in initiating and maintaining myelination of the central nervous system for almost one-third of patients with TC deficiency demonstrated developmental delay [1, 17]. Early diagnosis and treatment could alleviate and prevent permanent neurologic sequelae [1, 17]. A case report of a TC deficiency patient who exhibited urinary methylmalonic acid excretion at birth may indicate intrauterine onset of the metabolic derangement and neurologic damage of this intriguing disorder [18]. This may explain neurocognitive impairment in some patients despite receiving prompt diagnosis and treatment at birth [14].

In summary, TC deficiency should be suspected in infant with chronic pancytopenia, hepatosplenomegaly, metabolic acidosis of unclear cause, and recurrent infection with or without overt megaloblastic change.

## Data Availability

The datasets generated and/or analyzed during the current study are available in the ClinVar repository (accession number SCV001981507, https://www.ncbi.nlm.nih.gov/clinvar/).

## Abbreviations

ALPS: Autoimmune lymphoproliferative syndrome
ASD: Autistic spectrum disorder
BM: Bone marrow
CBC: Complete blood count
EN + PN: Enteral and parenteral nutrition
G6PD: Glucose 6 phosphate dehydrogenase
Hb: Hemoglobin
IBMFS: Inherited bone marrow failure syndrome
IM: Intramuscular
JMML: Juvenile myelomocytic leukemia
JP: Just palpable
LDH: Lactate dehydrogenase
MCV: Mean corpuscular volume
MDS: Myelodysplastic syndrome
ND: No data
NG: Nasogastric tube
PCR: Polymerase chain reaction
PEM: Protein energy malnutrition
PID: Primary immune deficiency
PJP: Pneumocystis jirovecii
RDW: Red blood cell distribution width
TC: Transcobalamin II
URI: Upper respiratory tract infection
WES: Whole exome sequencing
